# To Dr. Bradley

**Published:** 1803-05-01

**Authors:** Thomas Beddoes

**Affiliations:** Clifton


### To Dr. BRADLEY.

SIR,

A Good deal of communication with medical men about the Influenza, makes me apprehend that the next epidemic of this kind, will find our successors as uncertain in some essential respects as our predecessors have left us. One of the first points of view in which I regard every disorder, is its *prevention*; for, really, the description of the current epidemic is quite easy, and its treatment, upon the whole, a very common place medical problem.

To know how far the Influenza can be prevented, we ought to settle whether it be contagious. In 1782, I think the best and most approved authorities were for the affirmative; and very many practitioners, I know, infer the same now from their observations. On this supposition, acid fumigations bid fair to stop the progress of the complaint, and they have been used in families, without any inconvenience, to preserve the uninfected, though it might not always have been proper to fumigate the apartments of the sick. Their general employment *might* have determined the question concerning infection.

Those who ascribe the epidemic to the atmosphere, I see, rest their opinion upon exceptions. It is therefore of the utmost importance to state the proportions and character of the exceptions; that is, the number of instances where where only one in a family has suffered, and so on. The greatest care ought also to be taken, not to mistake *Catarrh* for *Influenza*. This can often with difficulty be done. At least many practitioners have said so in their correspondence with me. But the symptoms, together with the inquiry, *whether the patient had been in a situation to catch cold*, would frequently lead to a satisfactory conclusion.—It would also be very desirable to ascertain the number of persons affected, who had been carefully screened from the cold during the severe weather. Such cases were, I believe, quite common here.

Too little attention appears to me to have been paid to the following consideration. Exceptions to infection in measles, scarlatina, &c. are frequent enough even in the case of bed-fellows; and yet few of those who doubt in the case of the Influenza, become sceptical with regard to the contagious nature of the other disorders. One would therefore desire to know, how far the exceptions were more numerous or more remarkable in this epidemic.

I would further propose as a query, *whether the exceptions can be referred to particular ages, &c.* It would be no more extraordinary that the contagion of Inflnenza (supposing it to exist) should be less active on particular classes than the contagion of scarlet fever. By attending to these hints, which might be greatly extended, the Communications of your Correspondents may be more pointed and decisive; for simply to enumerate certain exceptions, comes to nothing. It might be proved in this manner that no disease is contagious.

I am, &c.

*Clifton, April* 19, 1803. THOMAS BEDDOES.

